# Bidirectional Relationship Between Body Pain and Depressive Symptoms: A Pooled Analysis of Two National Aging Cohort Studies

**DOI:** 10.3389/fpsyt.2022.881779

**Published:** 2022-04-26

**Authors:** Yujia Qiu, Yanjun Ma, Xuebing Huang

**Affiliations:** ^1^NHC Key Laboratory of Mental Health (Peking University), Clinical Research Center, Peking University Sixth Hospital, Peking University Institute of Mental Health, National Clinical Research Center for Mental Disorders (Peking University Sixth Hospital), Beijing, China; ^2^Peking University Clinical Research Institute, Peking University First Hospital, Beijing, China

**Keywords:** pain, depressive symptoms, nationally representative aging cohorts, prospective study, association between pain and depressive symptoms

## Abstract

**Aims:**

To investigate the bidirectional longitudinal association between pain and depressive symptoms and explore whether gender modifies the association.

**Methods:**

This study used data of 17,577 participants without depressive symptoms and 15,775 without pain at baseline from waves 1–8 (2002/2003 to 2016/2017) of the English Longitudinal Study of Aging (ELSA) and waves 1 to 3 [2011–2015] of the China Health Retirement Longitudinal Study (CHARLS). Cox regression models were performed at the cohort level to evaluate the potential longitudinal associations, and then random-effect meta-analyses were conducted to pool the results. The potential modifying effect was detected by *Z*-test.

**Results:**

During 103,512 person-years of follow-up in participants without depressive symptoms, baseline pain intensity was associated with incident depressive symptoms. Compared with individuals who reported no pain at baseline, the pooled adjusted hazard ratio (HR) of incident depressive symptoms for participants with mild to moderate pain and for those with severe pain was 1.37 (95% CI: 1.22–1.55, *p* < 0.001) and 1.52 (95% CI: 1.34–1.73, *p* < 0.001), respectively. During 81,958 person-years of follow-up in participants without pain, baseline depressive symptoms were associated with a significantly higher incidence of pain, and the pooled adjusted HR of incident pain was 1.71 (95% CI: 1.60–1.82, *p* < 0.001). These associations were not modified by gender.

**Conclusions:**

A bidirectional longitudinal association between pain and depressive symptoms was demonstrated, not modified by gender. Family doctors should be aware of the bidirectional association and advice individuals with pain or depressive symptoms to be screened for both kinds of symptoms.

## Introduction

Both pain and depression are considerable health-related concerns that draw increasing attention globally (1). As indicated by a study that covered 17 countries, nearly two-fifths of adults reported chronic pains during the previous year, and it is noteworthy that the figure is rising in line with the population aging (2). Depression is another age-related disease that affects 322 million individuals worldwide (3). The annual financial costs of pain and depression had been up to $560 billion and $210.5 billion in the US, respectively (4, 5). The comorbidity of these two diseases is quite common. Pain conditions were complained by more than half of depression patients (6). In turn, more than 50% of patients with chronic pain suffered from depressive symptoms (7). The pain in depressive patients affected the treatment and prognosis of depression and vice versa (7). Thus, a thorough understanding of the association between pain and depressive symptoms is necessary.

Most of the previous studies on the association between pain and depression used cross-sectional data or only researched unidirectional longitudinal association. There have been several prospective studies on the bidirectional relationship between pain and depression, while these studies yielded inconsistent conclusions and were limited by representativeness, follow-up duration, or other methodological pitfalls (1, 8–12). The evidence from community-based large cohorts is still lacking. Moreover, the gender difference in the potential bidirectional longitudinal association remains unclear.

The English Longitudinal Study of Aging (ELSA) (13) and the China Health Retirement Longitudinal Study (CHARLS) (14) are two nationally representative aging cohorts that involved pain and depressive symptoms at multiple waves. These two cohorts provided an opportunity to investigate the longitudinal associations between pain and depressive symptoms. Our aims were (1) to examine the bidirectional longitudinal association between pain and depressive symptoms in community-based aging populations and (2) to explore whether gender modifies the association.

## Materials and Methods

### Participants

The data were derived from the ELSA and the CHARLS, both of which are community-based and nationally representative aging cohorts. The ELSA and the CHARLS were approved by London Multicentre Research Ethics Committee and Peking University Institutional Review Board, respectively (13–15). Written informed consent forms were obtained from all participants in both cohorts.

The CHARLS cohort was of individuals who aged 45 years and older and were living in China (14). Multistage probability sampling was used in the CHARLS to ensure the nationally representativeness. The present study used the data from wave 1 (2011) as a baseline and considered waves 2–3 (2013–2015) as a follow-up period. The ELSA sample was from the Health Survey for England, which randomly enrolled individuals who aged 50 years or older and were living in England using the postcode (13, 16). The baseline of the ELSA cohort was wave 1 (2002/2003) and the follow-up waves included waves from 2 to 8 (2004/2005 to 2016/2017) (17).

**Figure 1:**
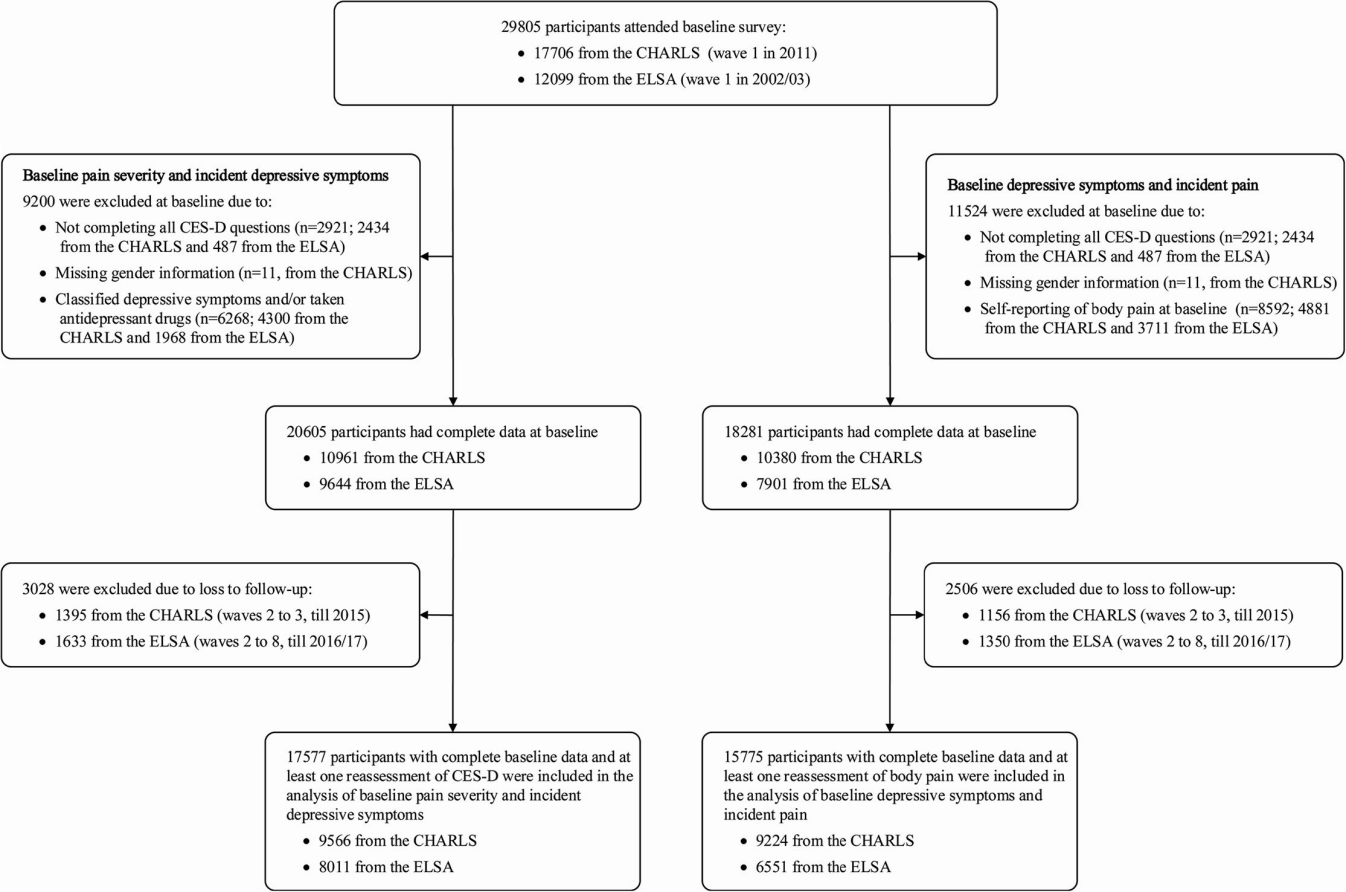
Flow chart of inclusion of this study population.

Figure 1 shows the flow chart of participant selection for the present study. In the analysis of the association between baseline pain intensity and incident depressive symptoms, 17,577 participants (9,566 from the CHARLS and 8,011 from the ELSA) free of depressive symptoms at baseline and with ≥1 remeasurement of depressive symptoms were included in the present study (Figure 1). To analyze the association between baseline depressive symptoms and incident pain, this study included 15,775 participants (9,224 from the CHARLS and 6,551 from the ELSA) free of pain at baseline and with ≥1 reassessment of pain (Figure 1).

### Assessments

#### Assessment of Pain

In both cohorts, pain intensity at baseline and each follow-up wave were assessed. In the CHARLS cohort, baseline pain intensity was categorized into three groups: no pain, mild to moderate pain, and severe pain. If there were more than one pain location, the most severe one among them was recorded. Fifteen specific common pain locations have been assessed in the CHARLS: head, shoulders, arms, wrist, fingers, chest, stomach, back, waist, buttocks, legs, knees, ankles, toes, and neck (18). In the ELSA cohort, pain intensity was measured based on pain intensity scores ranging from 0 to 10 (0 was no pain and 10 was severe or excruciating pain). Four specific pain locations have been evaluated in the ELSA: back, hips, knees, and feet (17). For pooling analyses in this study, we divided the ELSA participants into three categories of pain intensity according to the pain intensity scores: no (scored 0), mild to moderate (scored 1–7), and severe pain (scored 8–10). The cutoff points were established according to Boonstra et al. (19). The most severe one among the four locations was used in our analyses.

In the longitudinal association analysis of baseline pain and incident depressive symptoms, pain intensity was used as a potential risk factor. In turn, incident self-reported pain that includes mild to severe pain was used as the outcome when analyzing the association between baseline depressive symptoms and incident pain.

#### Assessment of Depressive Symptoms

Depressive symptoms were assessed by an eight-item version of the Center for Epidemiologic Studies Depression Scale (CES-D) in the ELSA (20). One point was given for each item, and the total scores were ranged from 0 to 8. According to previous studies, a score of 4 or more was defined as depressive symptoms (20, 21). The CHARLS used CES-D (ten-item version) to assess depressive symptoms (22). Three points were given for each item. Thus, the scores were ranged from 0 to 30. Depressive symptoms were defined as a score ≥12 according to prior validation studies (22, 23).

### Covariates

Covariates that were selected in our study include demographics (age and gender), socioeconomic status (education in years and cohabitation status), lifestyle factors (smoking and alcohol consumption), and relevant clinical characteristics (self-reported physician-diagnosed history of hypertension, diabetes, coronary heart disease, stroke, cancer, and chronic lung disease) (15). Education levels of ≥senior high school in the CHARLS and ≥level 1 national vocational qualification or General Certificate of Education Advanced level in the ELSA were defined as a high level of education (24). Cohabitation status was defined as currently living alone or not. Only current smokers were defined as smokers.

### Statistical Analysis

The results are presented as percentages for categorical variables and means ± standard deviation (SD) for continuous variables. Data were firstly analyzed at cohort level according to the following uniform protocol. We used Cox regression models to evaluate the relationship between baseline pain category and incident depressive symptoms and the association between baseline depressive symptoms and incident pain. After adjustment for the covariates mentioned above, hazard ratios (HRs) and corresponding 95% confidence intervals (CIs) were reported. Then, pooled analyses were performed to estimate the pooled effect and 95% CIs with random-effect meta-analyses, which took heterogeneity of the two cohorts into consideration. The *I*^2^ statistic was used to present the extent of variability between the two studies that were attributable to heterogeneity. To assess the potential modifying effects of gender on the bidirectional relationship, a *Z*-test was performed to compare the coefficients between two subgroup analyses, with the method proposed by Altman and Bland (25). To evaluate the stability of our main results, we performed longitudinal analyses of baseline pain and incident depressive symptoms repeatedly and restricted to participants with baseline CES-D scores ≤ 1 (median) in the ELSA and ≤ 5 (median) in the CHARLS to control the reverse causality.

Regression analyses were conducted using SAS software, version 9.4 (SAS Institute Inc., Cary, NC, USA). Meta-analyses were performed using STATA (version 11; Stata Corp, College Station, TX, USA). All analyses were two-sided with a threshold value of *p*- equals to 0.05 for statistical significance.

## Results

### Baseline Characteristics

A total of 17,577 individuals (9,566 from the CHARLS and 8,011 from the ELSA) without depressive symptoms at baseline were included in the pooled analysis of the association between pain intensity and incident depressive symptoms, and 15,775 participants free of pain (9,224 from the CHARLS and 6,551 from the ELSA) at baseline were included in this analysis of the association between depressive symptoms and incident pain. Table 1 presents the baseline characteristics of the two cohorts separately.

**Table 1:** Characteristics of participants in the China Health Retirement Longitudinal Study (CHARLS) and the English Longitudinal Study of Aging (ELSA) at baseline.

**Characteristics**	**Participants without depressive symptoms at baseline**		**Participants without pain at baseline**	
	**CHARLS (*n* = 9,566)**	**ELSA (*n* = 8,011)**	**CHARLS (*n* = 9,224)**	**ELSA (*n* = 6,551)**
Age (years)	57.8 ± 9.2	63.2 ± 10.3	58.2 ± 9.5	62.9 ± 10.5
Women (%)	4,656 (48.7)	4,357 (54.4)	4,491 (48.7)	3,554 (54.3)
Pain severity (%)				
No pain	7,460 (78.0)	5,827 (72.7)	9,224 (100.0)	6,551 (100.0)
Mild to moderate pain	1,465 (15.3)	1,564 (19.5)	0 (0.0)	0 (0.0)
Severe pain	641 (6.7)	620 (7.7)	0 (0.0)	0 (0.0)
Depressive symptoms (%)	0 (0.0)	0 (0.0)	1,554 (16.9)	724 (11.1)
CES-D scores^*^	5.1 ± 3.3	0.81 ± 0.97	6.6 ± 5.3	1.16 ± 1.70
High level of education (%)	1,427 (14.9)	2,631 (32.8)	1,349 (14.6)	2,264 (34.6)
Living alone (%)	864 (9.0)	2,310 (28.8)	990 (10.7)	2,022 (30.9)
Current smoking (%)	3,059 (32.0)	1,314 (16.4)	2,939 (31.9)	1,104 (16.9)
Alcoholic drink ≥once per week (%)	1,727 (18.1)	4,994 (62.3)	1,624 (17.6)	4,197 (64.1)
Hypertension (%)	2,176 (22.8)	2,842 (35.5)	2,031 (22.0)	2,222 (33.9)
Diabetes (%)	481 (5.0)	484 (6.0)	451 (4.9)	357 (5.5)
Coronary heart disease (%)	942 (9.9)	799 (10.0)	862 (9.4)	587 (9.0)
Stroke (%)	141 (1.5)	244 (3.1)	133 (1.4)	191 (2.9)
Cancer (%)	70 (0.7)	434 (5.4)	62 (0.7)	352 (5.4)
Chronic lung disease (%)	770 (8.1)	400 (5.0)	696 (7.6)	273 (4.2)
Asthma (%)	242 (2.5)	819 (10.2)	219 (2.4)	646 (9.9)

*The results are presented as mean ± SD or n (%)*.

* *The ranges of CES-D scores in the CHARLS and the ELSA were different*.

### Baseline Pain Intensity and Incident Depressive Symptoms

**Figure 2:**
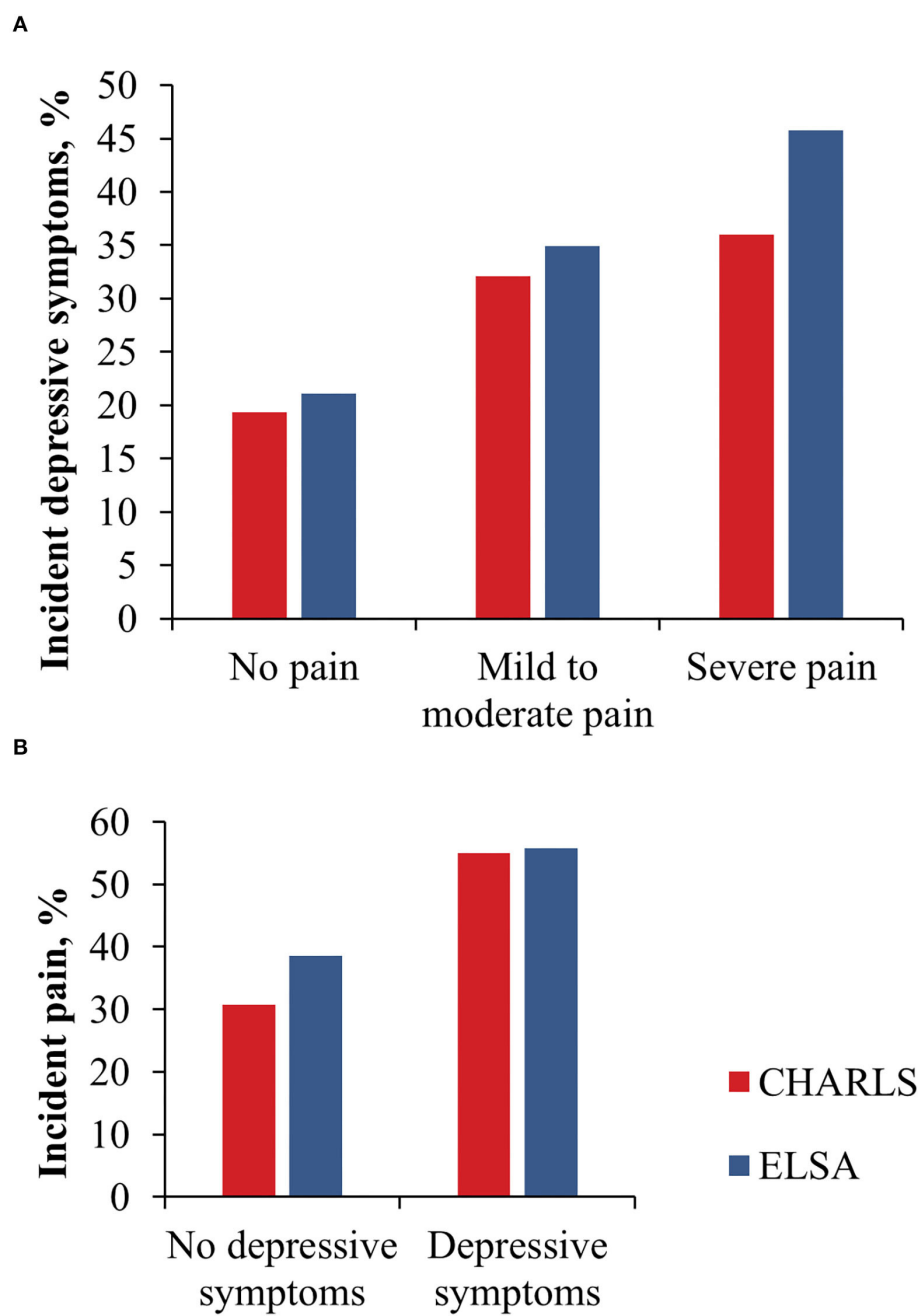
Incident depressive symptoms by baseline pain severity **(A)** and incident pain by baseline depressive symptoms **(B)**.

During 103,512 person-years of follow-up (the CHARLS: 33,586 and the ELSA: 69,926), we identified 2,140 participants (22.4%) in the CHARLS and 2,060 (25.7%) in the ELSA who have had incident depressive symptoms. The incidence of depressive symptoms was significantly increased by pain intensity in both cohorts (Figure 2 and Supplementary Table 1 in Supplementary File).

Compared with individuals who reported no pain at baseline, the pooled adjusted HR of incident depressive symptoms for participants with mild to moderate pain and for those with severe pain was 1.37 (95% CI: 1.22–1.55, *p* < 0.001) and 1.52 (95% CI: 1.34–1.73, *p* < 0.001), respectively (Table 2).

**Table 2:** Association between baseline pain severity and incident depressive symptoms, using Cox regression models.

**Pain category**	**CHARLS (*****n*** **=** **4,656)**		**ELSA (*****n*** **=** **4,357)**		**Meta-analysis (*****n*** **=** **9,013)**			
	HR (95% CI)^*^	*P* value^*^	HR (95% CI)^*^	*P* value^*^	**Pooled HR (95% CI)**	***P* value**	***I^**2**^* (%)**	***P* value**
No pain	Ref	/	Ref	/	Ref	/	/	/
Mild to moderate pain	1.29 (1.16–1.44)	<0.001	1.46 (1.32–1.62)	<0.001	1.37 (1.22–1.55)	<0.001	62.3	0.103
Severe pain	1.42 (1.23–1.64)	<0.001	1.62 (1.41–1.85)	<0.001	1.52 (1.34–1.73)	<0.001	41.3	0.192
Per category increase	1.22 (1.14–1.30)	<0.001	1.31 (1.23–1.39)	<0.001	1.27 (1.18–1.36)	<0.001	58.6	0.120

* *After adjusting for baseline CES-D scores, gender, age, education, marital status, current smoking, alcohol consumption, self-reported hypertension, diabetes, coronary heart disease, stroke, cancer, chronic lung disease, and asthma*.

### Baseline Depressive Symptoms and Incident Pain

During 81,958 person-years of follow-up (the CHARLS: 30,946 and the ELSA: 51,012), 3,209 individuals (34.8%) from the CHARLS cohort and 2,646 (40.4%) from the ELSA cohort reported incident pain. Compared with participants without depressive symptoms, individuals with baseline depressive symptoms at baseline had a significantly higher incidence of pain in both cohorts (Figure 2 and Supplementary Table 2 in Supplementary File).

Compared to participants without baseline depressive symptoms, those with depressive symptoms at baseline had a significantly higher incidence of pain in both cohorts after multivariable adjustment (pooled HR 1.71, 95% CI: 1.60–1.82, *p* < 0.001; Table 3).

**Table 3:** Association between baseline depressive symptoms and incident pain, using Cox regression model.

**Depressive symptoms**	**CHARLS (*****n*** **=** **9,224)**		**ELSA (*****n*** **=** **6,551)**		**Meta-analysis (*****n*** **=** **15,775)**			
	HR (95% CI)^*^;	*P* value^*^;	HR (95% CI)^*^;	*P* value^*^;	**Pooled HR (95% CI)**	***P* value**	***I^**2**^* (%)**	***P* value**
No	Ref	/	Ref	/	Ref	/	/	/
Yes	1.74 (1.61–1.89)	<0.001	1.65 (1.48–1.84)	<0.001	1.71 (1.60–1.82)	<0.001	0.0	0.441

* *After adjusting for gender, age, education, marital status, current smoking, alcohol consumption, self-reported hypertension, diabetes, coronary heart disease, stroke, cancer, chronic lung disease, and asthma*.

### Non-Response Analyses

Among the baseline population, 1,395 (12.7%) participants from the CHARLS and 1,633 (16.9%) from the ELSA with complete baseline data were excluded from the analyses on the association between baseline pain intensity and incident depressive symptoms for loss to follow-up. The excluded participants in both cohorts were older, had a higher percentage of living alone, smoking, coronary heart disease, self-reported diabetes, stroke, and chronic lung disease, and had a lower percentage of alcohol consumption (Supplementary Tables 3, 4 in Supplementary File). Similarly, individuals who were excluded from the analyses on the association between baseline depressive symptoms and incident pain also had higher levels of the major risk factors than those who were included in this study (data not shown).

### Subgroup Analyses by Gender

We performed repeated analyses by gender and found that the associations between baseline pain severity and incident depressive symptoms were still significant in both men and women (Supplementary Tables 5, 6 in Supplementary File). Table 4 shows the summarized results of pooled analyses by gender, and we found that these associations were not modified by gender.

**Table 4:** Modifying effects of sex on the association between baseline pain intensity and incident depressive symptoms, and on the association between baseline depressive symptoms and incident pain.

	Pooled HR (95% CI)^*^	***P* for interaction**
**Baseline pain intensity and incident depressive symptoms**		
No Pain	Ref	/
Mild to moderate pain		
Women	1.33 (1.09–1.62)	0.436
Men	1.46 (1.29–1.66)	
Severe pain		
Women	1.51 (1.25–1.83)	1.000
Men	1.51 (1.28–1.78)	
Per category increase		
Women	1.25 (1.12–1.41)	0.734
Men	1.28 (1.19–1.38)	
**Baseline depressive symptoms and incident pain**		
No depressive symptoms	Ref	/
Depressive symptoms		
Women	1.63 (1.48–1.80)	0.075
Men	1.86 (1.67–2.07)	

* *After adjusting for baseline CES-D scores (when analyzing the association between baseline pain intensity and incident depressive symptoms), age, education, marital status, current smoking, alcohol consumption, self-reported hypertension, diabetes, coronary heart disease, stroke, cancer, chronic lung disease, and asthma*.

### Sensitivity Analyses

We restricted analyses to participants with baseline CES-D scores ≤ 5 in the CHARLS and ≤ 1 in the ELSA. The results show that the associations between pain categories and incident depressive symptoms became stronger (Supplementary Table 7 in Supplementary File).

## Discussion

In this pooled study of two nationally representative aging cohorts, we detected a significant bidirectional longitudinal association between pain and depressive symptoms after adjustment for potential confounders. This bidirectional association was not modified by gender. To the knowledge of the authors, the present study is one the of largest cohort studies on this topic.

Our study provided additional evidence for the bidirectional association between pain and depression from community-based middle-aged and elderly people. Magni et al. firstly investigated and found the bidirectional relationship in a prospective study among patients with pain or depression in 1993, while they found the association was small in magnitude (8). Some short-term studies, which lasted only 1 or 2 years, found a bidirectional association between lower back pain and depression (9, 10, 26). Besides the limited follow-up period, these three studies were conducted among patients from outpatient or healthcare plans, thus the representativeness limited their power to demonstrate the association in the general population. In 2006, Chou analyzed the 2-year data of the ELSA and found the bidirectional longitudinal association between pain and depression (1). While this study did not perform a subgroup study according to gender and was limited by the short period, as Chou admitted (1). In a long-term cohort of 2,028 seniors, the bidirectional influence was also noticed but did not remain after adjustment for covariates (11). Recently, a large-sampled study proved a stronger bidirectional association between pain and mental illness than what we found (12). Nevertheless, this study only employed data from a register system, which not only caused selection bias but also limited its ability to investigate the temporal association between pain and depression, since patients with pain or depression did not necessarily go to see a doctor at the onset. Moreover, the registered study did not differentiate between depression and anxiety (12).

Some researchers suggested that female depression patients were more likely to be affected by chronic pain than male patients (27, 28). Besides, previous studies also observed that women were more sensitive to pain and pain-related distress in both real life and experiment (28–30). Thus, we assumed that gender might modify the bidirectional association between pain and depressive symptoms. However, our study did not find this modifying effect. It probably resulted from the age category, for all the participants who were above 45 in China and above 50 in England, around or after menopause. This population was nearly free from the effect of sex hormones. Consequently, future research could extend the population to younger individuals to investigate the potential gender difference of the bidirectional association between pain and depression.

The precise mechanisms that link pain and depression remain unclear. An adequate understanding of the bidirectional relationship and underlying mechanism may contribute to screening approach and prevention strategies for pain in patients with depressive symptoms and vice versa. The following potential mechanisms might explain the bidirectional relationship. It has been proved that neuroinflammation was of pivotal importance in the pathogenesis of both chronic pain and depression. High levels of corticosterone and cytokines induced microglial activation (31). This activation probably suppresses neurogenesis and neuroplasticity, leading to depressive symptoms (28, 32). Meanwhile, microglial activation has been identified as a key role in chronic pain development and maintenance (33). Thus, pain and depression probably share neuropathological mechanisms. Moreover, psychological factors that include self-perceived health-related quality of life might also mediate the bidirectional association between pain and depression (33).

The major strength of our study is the large long-term community-based cohorts. Both the ELSA and the CHARLS were nationally representative of two countries with substantial cultural differences and provided free premium multiwave data that cover depressive scores, pain severities, and covariates measurement. Second, this study detected bidirectional longitudinal influence, while most of the previous studies only explored across-sectional or unidirectional longitudinal association.

However, the presented study has several limitations. First, it is an observational study, which inevitably limited its ability to demonstrate the causal association. Thus, further mechanisms or experimental studies that focus on the bidirectional association are required. Second, over 10% of individuals were excluded in both cohorts due to loss to follow-up. These excluded individuals had higher levels of risk factors than those included in this study, which may limit the generalization of our results to the original populations (24). Third, it is noteworthy that the scales of pain intensity and depressive symptoms used in the ELSA and CHARLS were different. However, the consistent results under the different scales might be more persuasive than under a uniform scale. Fourth, pain and depressive symptoms used in this study were self-reported but not doctor-diagnosed, which might have biased our results. Fifth, current confounding might exist even though we have adjusted for a series of potential covariates. For example, the rural/urban differences in China might bias the bidirectional association between pain and depressive symptoms without a certain direction, while the consistency of the findings between the two cohorts indicated the magnitude of the bias might be small. Finally, there was small heterogeneity between the results of the ELSA and the CHARLS, which had been located as the influence of baseline depressive symptom scale on later incident pain for women.

## Conclusions

This pooled analysis of two national aging cohort studies provides further evidence that supports the bidirectional association between pain and depressive symptoms. Individuals with pain had a higher risk of incident depressive symptoms and vice versa. Gender does not modify the bidirectional association. This study suggested that family doctors should be aware of the bidirectional relationship and carry out early assessments or event interventions to reduce the possibility of depression in people with pain or to prevent pain in people with depressive symptoms. Future studies are necessary to explore the potential mechanisms underneath the bidirectional association.

## Data Availability Statement

Publicly available datasets were analyzed in this study. This data can be found here: http://charls.pku.edu.cn/; https://beta.ukdataservice.ac.uk/datacatalogue/series/series?id=2 00011.

## Ethics Statement

The studies involving human participants were reviewed and approved by London Multicentre Research Ethics Committee and Peking University Institutional Review Board. The patients/participants provided their written informed consent to participate in this study.

## Author Contributions

YQ and YM conducted the statistical analysis and drafted the original manuscript. XH conceived the study and revised the manuscript. All authors had final responsibility for the submission and all of them read and approved the final version of the manuscript.

## Funding

This study was supported by the Capital Health Research and Development of Special Fund Program (No. 2018-2-4114) and Self-exploration Project of National Clinical Research Center for Mental Disorders (No. NCRC2020Z05).

## Conflict of Interest

The authors declare that the research was conducted in the absence of any commercial or financial relationships that could be construed as a potential conflict of interest.

## Publisher's Note

All claims expressed in this article are solely those of the authors and do not necessarily represent those of their affiliated organizations, or those of the publisher, the editors and the reviewers. Any product that may be evaluated in this article, or claim that may be made by its manufacturer, is not guaranteed or endorsed by the publisher.
