# Supplementary material for: Bidirectional Relationship Between Body Pain and Depressive Symptoms: A Pooled Analysis of Two National Aging Cohort Studies
Source: Front Psychiatry. 2022 Apr 26;13:881779. doi: 10.3389/fpsyt.2022.881779 (PMC9086823; doi:10.3389/fpsyt.2022.881779)
Supplement: Supplementary file 1 [file Data_Sheet_1.docx]

**Supplementary File**

Bidirectional Relationship between Body Pain and Depressive Symptoms: A Pooled Analysis of Two National Ageing Cohort Studies

Yujia Qiu, Yanjun Ma, Xuebing Huang

**Supplementary Table 1. Incident depressive symptoms (%) during follow-up across baseline category of pain severity**

| **Pain severity** | **Incident depressive symptoms, n (%)** | | |
| --- | --- | --- | --- |
|  | **CHARLS (n=9566)** |  | **ELSA (n=8011)** |
| No pain | 1439 (19.3) |  | 1230 (21.1) |
| Mild to moderate pain | 470 (32.1) |  | 546 (34.9) |
| Severe pain | 231 (36.0) |  | 284 (45.8) |
| *P* for trend* | <0.001 |  | <0.001 |

*Calculated using a chi-square test for trend.

Supplementary Table 2. Incident pain (%) during follow-up by baseline depressive symptoms

| **Depressive symptoms** | **Incident pain, n (%)** | | |
| --- | --- | --- | --- |
|  | **CHARLS (n=9224)** |  | **ELSA (n=6551)** |
| No | 2355 (30.7) |  | 2242 (38.5) |
| Yes | 854 (55.0) |  | 404 (55.8) |
| *P* for difference* | <0.001 |  | <0.001 |

*Calculated using a chi-square test.

**Supplementary Table 3. Comparison of baseline characteristics between the CHARLS participants included (n=9566) and excluded due to loss to follow-up (n=1395), when analyzing the association between baseline pain severity and incident depressive symptoms**

| **Characteristic** | **Included**  **(n=9566)** | **Loss to follow-up (n=1395)** | ***P* for difference*** |
| --- | --- | --- | --- |
| Age (years) | 57.8±9.2 | 61.4±12.1 | <0.001 |
| Women (%) | 4656 (48.7) | 685 (49.1) | 0.763 |
| CES-D scores | 5.1±3.3 | 5.2±3.3 | 0.500 |
| High level of education (%) | 1427 (14.9) | 296 (21.2) | <0.001 |
| Living alone (%) | 864 (9.0) | 218 (15.6) | <0.001 |
| Current smoking (%) | 3059 (32.0) | 415 (29.8) | 0.095 |
| Alcoholic drink ≥once per week (%) | 1727 (18.1) | 202 (14.5) | 0.001 |
| Hypertension (%) | 2176 (22.8) | 376 (27.0) | <0.001 |
| Diabetes (%) | 481 (5.0) | 89 (6.4) | 0.034 |
| Coronary heart disease (%) | 942 (9.9) | 170 (12.2) | 0.007 |
| Stroke (%) | 141 (1.5) | 36 (2.6) | 0.002 |
| Cancer (%) | 70 (0.7) | 12 (0.9) | 0.603 |
| Chronic lung disease (%) | 770 (8.1) | 137 (9.8) | 0.025 |
| Asthma (%) | 242 (2.5) | 44 (3.2) | 0.172 |

The results are presented as mean ± SD, or n (%).

*The differences between participants included and excluded were tested using the *t*-test or chi-square test.

**Supplementary Table 4. Comparison of baseline characteristics between the ELSA participants included (n=8011) and excluded due to loss to follow-up (n=1633), when analyzing the association between baseline pain severity and incident depressive symptoms**

| **Characteristic** | **Included**  **(n=8011)** | **Loss to follow-up (n=1633)** | ***P* for difference*** |
| --- | --- | --- | --- |
| Age (years) | 63.2±10.3 | 66.6±12.7 | <0.001 |
| Women (%) | 4357 (54.4) | 840 (51.4) | 0.029 |
| CES-D scores | 0.81±0.97 | 0.94±1.04 | <0.001 |
| High level of education (%) | 2631 (32.8) | 370 (22.7) | <0.001 |
| Living alone (%) | 2310 (28.8) | 520 (31.8) | 0.015 |
| Current smoking (%) | 1314 (16.4) | 303 (18.6) | 0.034 |
| Alcoholic drink ≥once per week (%) | 4994 (62.3) | 893 (54.7) | <0.001 |
| Hypertension (%) | 2842 (35.5) | 616 (37.7) | 0.085 |
| Diabetes (%) | 484 (6.0) | 137 (8.4) | <0.001 |
| Coronary heart disease (%) | 799 (10.0) | 202 (12.4) | 0.004 |
| Stroke (%) | 244 (3.1) | 80 (4.9) | <0.001 |
| Cancer (%) | 434 (5.4) | 109 (6.7) | 0.045 |
| Chronic lung disease (%) | 400 (5.0) | 116 (7.1) | <0.001 |
| Asthma (%) | 819 (10.2) | 192 (11.8) | 0.065 |

The results are presented as mean ± SD, or n (%).

*The differences between participants included and excluded were tested using the *t*-test or chi-square test.

**Supplementary Table 5. Association between baseline pain severity and incident depressive symptoms by sex, analyzed using Cox regression models**

| **Pain category** | | **CHARLS (n=4656)** | |  | **ELSA (n=4357)** | |  | **Meta-analysis (n=9013)** | | | |
| --- | --- | --- | --- | --- | --- | --- | --- | --- | --- | --- | --- |
|  |  | **HR (95% CI)*** | ***P* value*** |  | **HR (95% CI)*** | ***P* value*** |  | **Pooled HR (95% CI)** | ***P* value** | ***I^2^* (%)** | ***P* value** |
| Female |  |  |  |  |  |  |  |  |  |  |  |
|  | No pain | Ref | / |  | Ref | / |  | Ref | / | / | / |
|  | Mild to moderate pain | 1.20 (1.05–1.37) | <0.001 |  | 1.47 (1.30–1.67) | <0.001 |  | 1.33 (1.09–1.62) | 0.005 | 78.9 | 0.029 |
|  | Severe pain | 1.37 (1.15–1.65) | <0.001 |  | 1.66 (1.41–1.96) | <0.001 |  | 1.51 (1.25–1.83) | <0.001 | 57.8 | 0.124 |
|  | Per category increase | 1.18 (1.09–1.28) | <0.001 |  | 1.33 (1.23–1.43) | <0.001 |  | 1.25 (1.12–1.41) | <0.001 | 77.9 | 0.033 |
| Male |  |  |  |  |  |  |  |  |  |  |  |
|  | No pain | Ref | / |  | Ref | / |  | Ref | / | / | / |
|  | Mild to moderate pain | 1.50 (1.25–1.78) | <0.001 |  | 1.43 (1.20–1.71) | <0.001 |  | 1.46 (1.29–1.66) | <0.001 | 0.0 | 0.708 |
|  | Severe pain | 1.49 (1.18–1.87) | <0.001 |  | 1.53 (1.20–1.94) | <0.001 |  | 1.51 (1.28–1.78) | <0.001 | 0.0 | 0.876 |
|  | Per category increase | 1.28 (1.15–1.42) | <0.001 |  | 1.28 (1.15–1.43) | <0.001 |  | 1.28 (1.19–1.38) | <0.001 | 0.0 | 1.000 |

*After adjusting for baseline CES-D scores, age, education, marital status, current smoking, alcohol consumption, self-reported hypertension, diabetes, coronary heart disease, stroke, cancer, chronic lung disease, and asthma.

**Supplementary Table 6. Association between baseline depressive symptoms and incident pain by sex, analyzed using Cox regression models**

| **Depressive symptoms** | | **CHARLS (n=4491)** | |  | **ELSA (n=3554)** | |  | **Meta-analysis (n=8045)** | | | |
| --- | --- | --- | --- | --- | --- | --- | --- | --- | --- | --- | --- |
|  |  | **HR (95% CI)*** | ***P* value*** |  | **HR (95% CI)*** | ***P* value*** |  | **Pooled HR (95% CI)** | ***P* value** | ***I^2^* (%)** | ***P* value** |
| Female |  |  |  |  |  |  |  |  |  |  |  |
|  | No | Ref | / |  | Ref | / |  | Ref | / | / | / |
|  | Yes | 1.70 (1.54–1.88) | <0.001 |  | 1.54 (1.35–1.75) | <0.001 |  | 1.63 (1.48–1.80) | <0.001 | 28.6 | 0.237 |
| Male |  |  |  |  |  |  |  |  |  |  |  |
|  | No | Ref | / |  | Ref | / |  | Ref | / | / | / |
|  | Yes | 1.83 (1.60–2.08) | <0.001 |  | 1.91 (1.58–2.31) | <0.001 |  | 1.86 (1.67–2.07) | <0.001 | 0.0 | 0.716 |

*After adjusting for baseline age, education, marital status, current smoking, alcohol consumption, self-reported hypertension, diabetes, coronary heart disease, stroke, cancer, chronic lung disease, and asthma.

**Supplementary Table 7. Sensitivity analysis: association between baseline pain severity and incident depressive symptoms by sex, among participants with baseline CES-D scores ≤5 in the CHARLS and ≤1 in the ELSA**

| **Pain category** | | **CHARLS (n=4656)** | |  | **ELSA (n=4357)** | |  | **Meta-analysis (n=9013)** | | | |
| --- | --- | --- | --- | --- | --- | --- | --- | --- | --- | --- | --- |
|  |  | **HR (95% CI)*** | ***P* value*** |  | **HR (95% CI)*** | ***P* value*** |  | **Pooled HR (95% CI)** | ***P* value** | ***I^2^* (%)** | ***P* value** |
| Female |  |  |  |  |  |  |  |  |  |  |  |
|  | No pain | Ref | / |  | Ref | / |  | Ref | / | / | / |
|  | Mild to moderate pain | 1.20 (0.93–1.55) | 0.152 |  | 1.50 (1.27–1.76) | <0.001 |  | 1.40 (1.22–1.61) | <0.001 | 49.5 | 0.159 |
|  | Severe pain | 1.32 (0.90–1.93) | 0.158 |  | 1.78 (1.39–2.28) | <0.001 |  | 1.63 (1.32–2.00) | <0.001 | 40.3 | 0.196 |
|  | Per category increase | 1.17 (0.99–1.37) | 0.061 |  | 1.38 (1.24–1.54) | <0.001 |  | 1.31 (1.20–1.43) | <0.001 | 66.0 | 0.086 |
| Male |  |  |  |  |  |  |  |  |  |  |  |
|  | No pain | Ref | / |  | Ref | / |  | Ref | / | / | / |
|  | Mild to moderate pain | 1.68 (1.22–2.32) | 0.001 |  | 1.59 (1.27–1.99) | <0.001 |  | 1.62 (1.35–1.95) | <0.001 | 0.0 | 0.773 |
|  | Severe pain | 2.05 (1.33–3.16) | 0.001 |  | 1.86 (1.28–2.71) | 0.001 |  | 1.94 (1.46–2.57) | <0.001 | 0.0 | 0.737 |
|  | Per category increase | 1.50 (1.24–1.80) | <0.001 |  | 1.44 (1.24–1.68) | <0.001 |  | 1.46 (1.30–1.65) | <0.001 | 0.0 | 0.768 |

*After adjusting for baseline CES-D scores, age, education, marital status, current smoking, alcohol consumption, self-reported hypertension, diabetes, coronary heart disease, stroke, cancer, chronic lung disease, and asthma.
